# Pancreaticoduodenectomy with right hemicolectomy for advanced malignancy: a single UK hepatopancreaticobiliary centre experience

**DOI:** 10.1111/codi.16303

**Published:** 2022-09-01

**Authors:** Bibek Das, Matyas Fehervari, Sahar Hamrang‐Yousefi, Long R. Jiao, Madhava Pai, John T. Jenkins, Duncan R. C. Spalding

**Affiliations:** ^1^ Department of Surgery and Cancer, Faculty of Medicine Imperial College London London UK; ^2^ Department of Hepatopancreaticobiliary Surgery Hammersmith Hospital London UK; ^3^ Department of Surgery St Mark's Hospital London UK

**Keywords:** colectomy, colonic neoplasms, extended pancreatectomy, hemicolectomy, pancreatic neoplasms, pancreaticoduodenectomy

## Abstract

**Aim:**

Locally advanced intestinal neoplasms including colon cancer may require radical *en bloc* pancreaticoduodenectomy and right hemicolectomy (PD‐RC) to achieve curative, margin‐negative resection, but the safety and benefit of this uncommon procedure has not been established. The Association of Coloproctology of Great Britain and Ireland IMPACT initiative has also highlighted a lack of awareness about current services available within the UK for patients with advanced colorectal cancer and concerns about low‐volume centres managing complex cases. Thus, we aimed to review the feasibility, safety and long‐term outcomes of this procedure at a single high‐volume hepatopancreaticobiliary surgery unit in the UK.

**Method:**

A retrospective cohort study was performed using a database of all consecutive patients with intestinal cancer who had been referred to our regional advanced multidisciplinary team and undergone PD‐RC in a 7‐year period (2013–2020). Clinico‐pathological and outcome data were reviewed.

**Results:**

Ten patients (mean age 54 ± 13, 8/10 men) were identified. Final histology revealed the primary tumour sites were colon (*n* = 7) and duodenum (*n* = 3). R0 resection was achieved in all cases. The major complication rate (Clavien–Dindo ≥ 3) was 10% (1/10) with no deaths within 90 days of surgery. The Kaplan–Meier estimated 5‐year overall survival was 83.3% (95% CI 58.3%–100%). Univariate survival analysis identified perineural invasion and extra‐colonic origin as predictors of poor survival (log‐rank *P* < 0.05).

**Conclusion:**

*En bloc* PD‐RC for locally advanced intestinal cancer can be performed safely with a high proportion of margin‐negative resections and resultant long‐term survival in carefully selected patients.

**What does this paper add to the literature?**

- The safety and benefit of pancreaticoduodenectomy with right hemicolectomy (PD‐RC) for locally advanced intestinal cancer has not been established.
- No studies have reported on the outcomes of PD‐RC for intestinal cancer in the UK.
- Good oncological outcomes can be achieved after PD‐RC, particularly for primary colon cancers.

## BACKGROUND

Locally advanced intestinal neoplasms presenting with colo‐duodenal fistulas may require radical *en bloc* pancreaticoduodenectomy (PD) and right hemicolectomy (RC) to achieve complete (R0) resection. Malignant colo‐duodenal fistulas may be secondary to either advanced colon cancer or, more rarely, primary duodenal cancer [1, 2]. Both PD and RC in isolation, however, remain major surgical procedures with considerable morbidity and mortality. The complication rate of PD can exceed 40% even in experienced hands, with postoperative pancreatic fistula (POPF) being a major determinant of short‐term outcomes [3]. Although RC may have lower rates of complications, the overall mortality is still estimated at 2%–3%, with colonic anastomotic leak as the key determinant of short‐term outcomes [4, 5].

Centralization of high‐risk operations to high‐volume centres has been associated with improved clinical outcomes [6]. For PD for cancer, centralization has been associated with both short‐ and long‐term improvements in the quality of cancer surgery and perioperative care. This includes an increase in margin‐negative (R0) resections, higher nodal clearance, fewer overall complications and improved management of post‐pancreatectomy complications [3, 7, 8]. These improvements, in turn, have been associated with a reduction in postoperative mortality to <5% and improved overall survival [7, 9]. The addition of RC to PD increases the complexity of the procedure and adds another anastomosis which, in theory, may increase the morbidity and mortality. This was suggested by a previous review of multivisceral resections with PD which showed a substantial increase in mortality (3‐fold) and morbidity compared to a standard PD [10]. With increasing surgical and institutional expertise in complex pancreatic resections at high‐volume units, however, the outcomes for multivisceral cancer resections in carefully selected patients may be equivalent to single‐organ resections.

Combined PD‐RC is an uncommon operation and a recent systematic review of the literature has reported that overall outcomes following PD with colonic resections are poor, with a morbidity of 12%–65% and surgery‐related mortality of 10% [11]. Primary tumour histology was a critical determinant of oncological outcomes in this review, as patients with well‐differentiated colonic adenocarcinomas without regional lymph node metastases had the best overall survival. In addition, previous reports have shown that curative multivisceral resection for colorectal cancer (CRC) is similar to standard resection [12]. The majority of cohorts included in this review, however, were from low/medium‐volume centres and no studies have reported on the feasibility, safety and outcomes of elective and emergency PD‐RC in a UK setting.

The Association of Coloproctology of Great Britain and Ireland has recently highlighted the priorities for advanced CRC patients after engaging with patient groups and clinical stakeholders through the Improving the Management of Patients with Advanced Colorectal Tumours (IMPACT) initiative [13]. This was in response to the considerable variation in the surgical management of these patients and lack of evidence from high‐quality clinical trials. A major concern was the lack of clarity and awareness about current services available within the UK for patients with advanced CRC and concerns about low‐volume centres managing complex cases. Our unit is a tertiary referral centre for hepatopancreaticobiliary (HPB) cancer surgery in England with an annual caseload of >50 PD, which can therefore be defined as a ‘high‐volume’ institution [4, 12]. Our primary objective was to review the management and clinical outcomes of all patients undergoing PD‐RC in our region in the UK over a 7‐year period in line with the themes of the IMPACT initiative.

## METHODS

A retrospective cohort study was performed using a prospectively maintained database of all consecutive adult patients (age >18 years) who had undergone PD‐RC in a 7‐year period (January 2013 to April 2020) at Hammersmith Hospital HPB Surgery Unit (London, UK) for intestinal cancer. The study has been reported in accordance with the STROBE guidelines (Table S1) [14]. All cases had been referred to the St Mark's Hospital (London, UK) CRC multidisciplinary team (MDT) meeting and additionally discussed at the Hammersmith Hospital pancreatic MDT meeting comprising surgeons, oncologists, gastroenterologists, pathologists and radiologists. Cases were referred either internally or externally from local colorectal MDTs within the northwest London network.

Preoperative imaging, including CT or MRI, was performed for staging. Staging was performed using the American Joint Committee on Cancer TNM classification. Additionally, upper gastrointestinal (GI) endoscopy with visualization of D2/D3 was performed in all cases to evaluate the extent of the duodenal involvement. Endoscopic ultrasound was used selectively to assess the degree of organ invasion to aid operative planning when imaging was not conclusive.

Patients were selected for surgery using the general criteria described in the National Institute for Health and Care Excellence, European Society for Medical Oncology and National Comprehensive Cancer Network guidelines for locally advanced non‐metastatic colon cancer: an absence of major pre‐existing conditions that would prevent major surgery, an absence of metastatic disease on staging investigations and the technical feasibility of a complete, margin‐negative *en bloc* resection of the tumour. A preoperative assessment was performed for all patients by a consultant anaesthetist to confirm fitness for major surgery.

Neoadjuvant chemotherapy (NAC) was offered to patients on a selective basis when the resection margin was deemed to be at risk, based on an MDT assessment of preoperative CT imaging. This was based on data from the FOxTROT trial which showed significantly lower margin involvement after NAC for locally advanced CRC [15].

The preliminary decision to perform a PD‐RC was made after MDT review of the histology, CT imaging and upper GI endoscopy ± endoscopic ultrasound findings. Radical *en bloc* resection (PD‐RC) was recommended for locally advanced tumours which were invading the second part of the duodenum (and/or the pancreatic head directly) adjacent to the ampulla of Vater and distal common bile duct. This would ensure wide margins and the greatest chance of a curative R0 resection. Pancreas‐preserving duodenal resections would only be recommended in cases where the duodenal involvement was limited and distant from the ampulla, and there was no direct involvement of the pancreas. The decision to perform a PD‐RC was then finalized after intra‐operative assessment of the degree of organ invasion by the operating surgeons.

All operations were performed using an open approach by experienced consultant HPB and colorectal surgeons. The procedure of choice was *en bloc* pylorus‐preserving PD with standard lymphadenectomy [16] and conventional lateral‐to‐medial RC. Reconstruction consisted of an end‐to‐side pancreatico‐jejunostomy, end‐to‐side hepaticojejunostomy, end‐to‐side duodenojejunostomy and a side‐to‐side ileo‐colic anastomosis. Postoperative complication severity was graded using the Clavien–Dindo classification [17] and pancreatectomy complications were recorded using the International Study Group of Pancreatic Surgery classifications [18, 19, 20]. Histology was reviewed by a consultant histopathologist with specialist experience in GI malignancy. In the case of any disagreement, the histology was reviewed by a second consultant histopathologist. After discharge, patients were followed up at 6–8 weeks after surgery and then at 3–6‐month intervals. Adjuvant chemotherapy or targeted therapy was offered to patients on a selective basis after histopathology review and MDT discussion.

Data on patient demographics, staging, treatment, clinical outcomes and overall survival were retrieved from the database. Categorical clinico‐pathological data were compared using a two‐tailed Fisher's exact test. Overall survival was calculated using the Kaplan–Meier method and survival outcomes were compared using the log‐rank test. All statistical analyses were conducted using SPSS® version 27 (IBM).

## RESULTS

**FIGURE 1:**
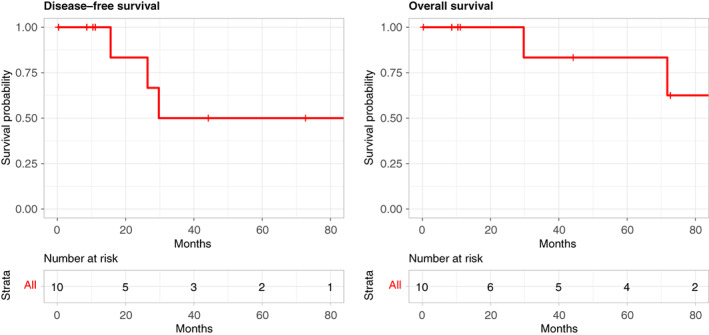
Kaplan–Meier survival plots for overall and disease‐free survival following PD‐RC.

Over a 7‐year period, 10 patients (mean age 54 ± 13, 8/10 men) underwent *en bloc* PD‐RC for cancer at our unit (Table 1). Most patients (90%; 9/10) were American Society of Anesthesiologists Grade 2. Complete (R0) resection was achieved in all cases. On final histology, the primary tumour site was the colon in seven patients (ascending colon and caecum three, hepatic flexure four) and duodenum (D2/D3) in three patients. Fistulation into the adjacent organ by adenocarcinoma was confirmed histologically in all cases (T4b) and in one case (patient 10) both a fistulating duodenal primary and a synchronous early transverse colon tumour were identified. Four patients initially received NAC comprising 5‐fluorouracil with a platinum agent. The median lymph node yield was 47 (range 15–74 lymph nodes), with regional lymph node metastases identified in 60% (6/10). Most patients had only minor complications (Clavien–Dindo < 3), with Grade B POPF reported in two patients which required parenteral nutrition and extended drainage only. One patient had recurrent upper GI bleeding secondary to an ulcer adjacent to the duodenojejunal anastomosis (Grade C post‐pancreatectomy haemorrhage) which required endoscopy and clipping (Clavien–Dindo Grade 3b). There were no colon‐related complications such as anastomotic leak or abscess. The median length of stay was 18 days (range 8–52 days) and no deaths were recorded within 90 days of surgery. Four patients received adjuvant chemotherapy. One patient (patient 3) with CRC developed local tumour recurrence 15 months after surgery in the perinephric bed and underwent further surgery. One additional patient with CRC (patient 1) developed lung metastases 26 months after surgery and underwent chemotherapy. Overall, the median length of follow‐up was 37 months (range 0.3–84.8 months) and there were two deaths. The Kaplan–Meier estimated 5‐year disease‐free survival rate and overall survival rate were 50% (95% CI 22.5%–100%) and 83.3% (95% CI 58.3%–100%) respectively (Figure 1).

**TABLE 1:** Summary of clinico‐pathological characteristics

Patient no.	Age	Gender	Alerting symptoms	Tumour site on final histology	Type of surgery	Chemotherapy	Lymph node metastases (LNR)	Differentiation	Maximum diameter (mm)	Resection status	Vascular invasion	Perineural invasion	MMR loss	Postoperative complication	Clavien–Dindo Grade	Last follow‐up (months since surgery)	Status
1	49	M	Abdominal pain	Colon: ascending colon	PPPD and right colectomy	Neoadjuvant	0/15 (0%)	Moderate	70	R0	No	No	Not tested	Mild pancreatitis	2	71.8	Deceased
2	42	M	Abdominal pain	Colon: hepatic flexure	PPPD, right colectomy, liver resection (segment VI) and reversal ileostomy	Adjuvant	4/48 (8.3%)	Poor	70	R0	Yes	No	Not tested	None	0	84.7	Alive
3	33	M	Abdominal pain	Colon: caecum	PPPD, right colectomy and small bowel resection	None	0/32 (0%)	Moderate	120	R0	No	No	Y	Grade A DGE	2	84.8	Alive
4	60	F	N&V	Colon: ascending colon	PPPD and right colectomy	None	0/57 (0%)	Poor	40	R0	No	No	Y	Grade C PPH	3b	72.7	Alive
5	71	M	N&V, abdominal pain, weight loss	Duodenum	PPPD and right colectomy	Adjuvant	1/39 (2.6%)	Poor	30	R0	Yes	Yes	Not tested	Chyle leak	2	29.7	Deceased
6	64	M	Abdominal pain, weight loss	Colon: hepatic flexure	PPPD and right colectomy	Neoadjuvant	0/45 (0%)	Moderate	60	R0	Extramural venous invasion	No	N	Grade B POPF	2	44.2	Alive
7	65	M	Abdominal pain	Duodenum	PPPD and right colectomy	Neoadjuvant	1/38 (2.6%)	Poor	25	R0	Yes	Yes	Not tested	None	0	(0.3)^a^	Alive
8	51	M	Abdominal pain, haematochezia	Colon: hepatic flexure	PPPD and right colectomy	Adjuvant	4/59 (6.8%)	Moderate	55	R0	Extramural venous invasion	Yes	Y	Mild pancreatitis	2	10.4	Alive
9	67	F	N&V, weight loss	Duodenum^b^	PPPD and right colectomy	Adjuvant	4/70 (5.7%)	Moderate	45	R0	Yes	Yes	Y	None	0	11.1	Alive
10	43	M	Abdominal pain	Colon: hepatic flexure	PPPD, right colectomy, partial hepatectomy, omentectomy, partial anterior wall resection and resection of 3 right anterior ribs	Neoadjuvant	1/74 (1.4%)	Poor	118	R0	No	No	N	Grade B POPF	2	8.6	Alive

Abbreviations: DGE, delayed gastric emptying; LNR, lymph node ratio; MMR, mismatch repair; N&V, nausea and vomiting; POPF, postoperative pancreatic fistula; PPH, post‐pancreatectomy haemorrhage; PPPD, pylorus‐preserving pancreaticoduodenectomy.

^a^ Incidental synchronous early transverse colon tumour.

^b^ Patient returned to country of origin with no further follow‐up.

Table 2 shows univariate analysis for overall survival. Patients with duodenal tumours on final histology had significantly worse overall survival than patients with a primary colonic tumour (*P* = 0.025; Figure S1). Perineural invasion was also associated with significantly worse overall survival. There were no significant associations between age, NAC or lymph node status and overall survival. No patients with duodenal tumours survived more than 3 years. Table 3 shows associations between tumour site and histological markers of lymphatic, vascular and perineural invasion. Duodenal tumours tended to have a higher rate of perineural invasion (*P* = 0.03).

**TABLE 2:** Univariate analysis of survival outcomes

		*n*	Number of survivors			Log‐rank *P* value
			1 year	3 year	5 year	
Age	≥60	4	2	1	0	0.16
	<60	6	4	4	4	
Gender	Female	2	1	1	1	0.47
	Male	8	5	4	3	
Site of tumour on final histology	Colon	7	5	5	4	0.0250*
	Duodenum	3	1	0	0	
Neoadjuvant therapy	No	6	4	3	3	0.52
	Yes	4	2	2	1	
Lymph node metastasis	Absent	4	4	4	3	0.52
	Present	6	2	1	1	
Differentiation	Moderate	5	3	3	2	1
	Poor	5	3	2	2	
Maximum tumour size (mm)	≥45	7	4	4	3	0.52
	<45	3	2	1	1	
Vascular invasion	No	4	3	3	3	0.71
	Yes	6	3	2	1	
Perineural invasion	No	6	5	5	4	0.025*
	Yes	4	1	0	0	
Resection status	R0	4	2	1	0	0.16
	R1/2	6	4	4	4	

* *p* < 0.05.

**TABLE 3:** Relationship between origin of tumour and markers of invasion

		Colonic	Duodenal	*P*
Lymph node metastasis	Present	3	3	0.20
	Absent	4	0	
Vascular invasion	Present	3	3	0.20
	Absent	4	0	
Perineural invasion	Present	1	3	0.03*
	Absent	6	0	

* *p* < 0.05.

Pancreaticoduodenectomy with right hemicolectomy was performed on an urgent basis for one patient (patient 10). This patient presented initially with large bowel obstruction secondary to a locally advanced mass at the hepatic flexure of the colon invading the liver, gallbladder, duodenum and chest wall (Figure S2). Initial biopsy confirmed a poorly differentiated adenocarcinoma. Imaging revealed no signs of distant metastatic disease. After being managed initially with endoluminal stenting and chemotherapy, he went on to develop gastric outlet obstruction and was transferred to our unit as an emergency for definitive treatment. The patient underwent radical surgery with curative intent, which involved PD, extended RC, segment V/VI liver resection, cholecystectomy, excision of anterior abdominal wall (including first three ribs), excision of right hemidiaphragm and wedge resection of superior mesenteric vein. Final pathology revealed that the tumour was 118 mm in maximum diameter, had negative margins (R0 resection), 1/74 lymph nodes were positive, and was a poorly differentiated colonic adenocarcinoma. The postoperative stay was complicated by a Grade B POPF managed with parenteral nutrition, antibiotics and extended drainage. The length of stay was 24 days, and the patient was alive without evidence of recurrence at 9 months follow‐up.

## DISCUSSION

We have reviewed the clinical outcomes of our 7‐year experience of performing *en bloc* PD‐RC in patients with locally advanced malignancy. We have shown that, out of 10 patients who underwent PD‐RC, all had negative margins (R0 resection) and experienced only minor complications (Clavien–Dindo Grade < 3b); long‐term (>5‐year) survival can be achieved.

Resection margin quality is a major indicator of surgical quality for pancreatic surgery units [21] and it is significant that all cases had a negative margin despite requiring *en bloc* multivisceral resection of large, locally advanced tumours. This is also clinically significant, since achieving margin‐negative resection in locally advanced tumours in multivisceral resection has been shown to produce similar survival to cases where there has been no adjacent organ involvement in stage‐matched patients [22].

The postoperative morbidity was also low as the rate of major complications (Clavien–Dindo ≥ 3) was only 10% (1/10). The rate of Grade B POPF was 20% (2/10) which is similar to reported POPF rates in the literature after PD and PD‐RC [11, 23]. There were no colonic anastomotic leaks in this series whereas previously reported rates after PD‐RC were as high as 33% [11]. No patients in this series required reoperation or died within 90 days of surgery which confirms that this procedure can be performed safely in experienced hands. Birkmeyer and others have previously noted that there is a strong inverse relationship between adverse clinical outcomes, specifically postoperative mortality, and hospital volume in relation to complex cancer surgery [24, 25]. This evidence has paved the way for centralization of complex operations such as PD to regional centres of excellence. Current evidence suggests a large part of the reduction in mortality at high‐volume hospitals can be attributed to a reduction in ‘failure to rescue’ scenarios [8, 26]. This requires robust institutional frameworks to recognize and react to complications after specialist surgery. All PD‐RC patients at our unit are therefore admitted to a specialist surgical intensive care unit after surgery where there is 24‐h specialist surgery (HPB and colorectal), endoscopy and interventional radiology expertise available. Consequently, the one major complication in our case series (post‐pancreatectomy upper GI haemorrhage) was managed promptly out‐of‐hours with specialist endoscopy and intensive care unit support. Thus, advanced CRC which may require complex surgery such as PD‐RC should be referred by local MDTs to a dedicated advanced CRC MDT at specialist units involving all key clinical stakeholders. This ensures that surgery takes place at a high‐volume centre after coordinated discussion between specialists.

Factors identified in this series associated with worse prognosis on univariate survival analysis were perineural invasion and extra‐colonic origin of the primary tumour on final histology. Pancreatico‐duodenal tumours are known to be biologically more aggressive tumours than colonic tumours, with a greater rate of metastasis and therapy resistance [27]. Nodal status was not an independent factor affecting prognosis as in other series [11], but this is probably due to the heterogeneous case mix (e.g., cancer type and use of systemic therapy).

The highlighted case (patient 10) also demonstrates that radical single‐stage multivisceral resection with negative margins can be performed safely on an urgent basis after referral to a specialist unit. Previous reports of emergency PD‐RC have shown that early and long‐term outcomes can be similar to those of non‐emergency PD‐RC when performed by experienced surgeons [28].

The role of neoadjuvant therapy versus upfront PD‐RC surgery has not been thoroughly evaluated in this setting due to the rarity of this presentation, but it would seem reasonable to consider downsizing tumours where the resection margin may be at risk. Although currently not recommended in international guidelines, previous clinical trials such as FOxTROT have shown potential benefit from NAC in increasing the likelihood of margin‐negative resection and overall survival, compared to a direct‐to‐surgery approach, in locally advanced CRC [15]. It is unknown, however, whether NAC could downstage tumours sufficiently to avoid the potential morbidity of PD‐RC altogether. Furthermore, a recent meta‐analysis found previous trials of NAC were limited by a lack of randomization and heterogeneity in NAC regimens [29]. Therefore, patient selection and choice of NAC for locally advanced CRC prior to PD‐RC is an important area of future research.

It is interesting to note that at least half of the tumours in this series had defective DNA mismatch repair (MMR), compared to an overall frequency of 15% among sporadic colonic tumours [30]. It is known that most sporadic MMR tumours occur in the right side of the colon which present later than left‐sided tumours [31], and hence colonic adenocarcinoma requiring PD‐RC may be more likely to harbour defective DNA MMR. While MMR‐deficient CRC may have a more favourable stage‐matched prognosis compared to MMR‐proficient tumours [32], they are also relatively resistant to 5‐fluorouracil‐based chemotherapy [33]. This may, in turn, affect the choice of and decision to administer neo(adjuvant) chemotherapy.

Although all PD‐RC procedures in this case series were performed using an open approach, there has been a growing trend to perform PD using a minimally invasive approach (laparoscopic or robot‐assisted) which may further reduce morbidity [34]. A totally laparoscopic technique to perform PD‐RC has been described recently [35]. However, there is currently a lack of high‐quality data confirming the safety or oncological superiority of a minimally invasive approach over an open approach for PD [36, 37]; therefore this procedure remains under investigation at pancreatic surgery units with extensive experience in minimally invasive surgery. Future studies may also reveal the utility of extended colonic resection (complete mesocolic excision and D3 lymphadenectomy) combined with PD in improving clinical outcomes for locally advanced CRC. The oncological superiority and safety of this approach for CRC is still under investigation but low‐quality evidence from a recent systematic review appears to suggest that better overall and disease‐free survival can be achieved with a more radical approach, particularly for more advanced (stage 2 and 3) CRC [38].

The main limitations of this work are the small sample size and case mix heterogeneity from a single centre which may limit the generalizability of the results. The good postoperative outcomes may, in part, be attributed to the fact that the cohort was carefully selected and relatively young without major cardiovascular comorbidities. A future multicentre national audit of PD‐RC practices and outcomes could reveal additional information on volume–outcome relationships and regional differences in practice (e.g. referral criteria, use of NAC, patient selection criteria) that could help inform and standardize practice nationally.

In conclusion, in a UK setting, *en bloc* PD‐RC for locally advanced intestinal cancer can be performed safely with a high proportion of margin‐negative resections and good long‐term outcomes. Due to the rarity and complexity of this procedure, regional referral networks with standardized referral criteria to advanced MDTs at specialist units may be required for PD‐RC and other types of multivisceral resection. This is based on the established relationship between volume and outcome in other complex surgical procedures. Pooling patients at high‐volume centres could also aid recruitment to clinical trials and improve access to experimental therapies. Further research is needed to determine optimum patient and tumour characteristics for this procedure and better define the role of neoadjuvant therapy.

## AUTHOR CONTRIBUTIONS

B.D., M.F. and S.H.Y. contributed to data collection, data analysis and drafting the paper. M.P., L.R.J., J.T.J. and D.R.C.S. contributed to conception and design, critical revision of the paper, and approval of the paper.

## FUNDING INFORMATION

None.

## CONFLICT OF INTEREST

None declared.

## ETHICAL STATEMENT

As a retrospective service evaluation, NHS REC review was not required.

## Supporting information


Figure S1

Figure S2

Table S1

## Data Availability

All data generated or analysed during this study are included in this published article (and its supplementary information files).
