# Supplementary material for: Pancreaticoduodenectomy with right hemicolectomy for advanced malignancy: a single UK hepatopancreaticobiliary centre experience
Source: Colorectal Dis. 2022 Sep 1;25(1):16–23. doi: 10.1111/codi.16303 (PMC10087186; doi:10.1111/codi.16303)
Supplement: Supplementary file 1 — Figure S1 [file CODI-25-16-s002.pdf]

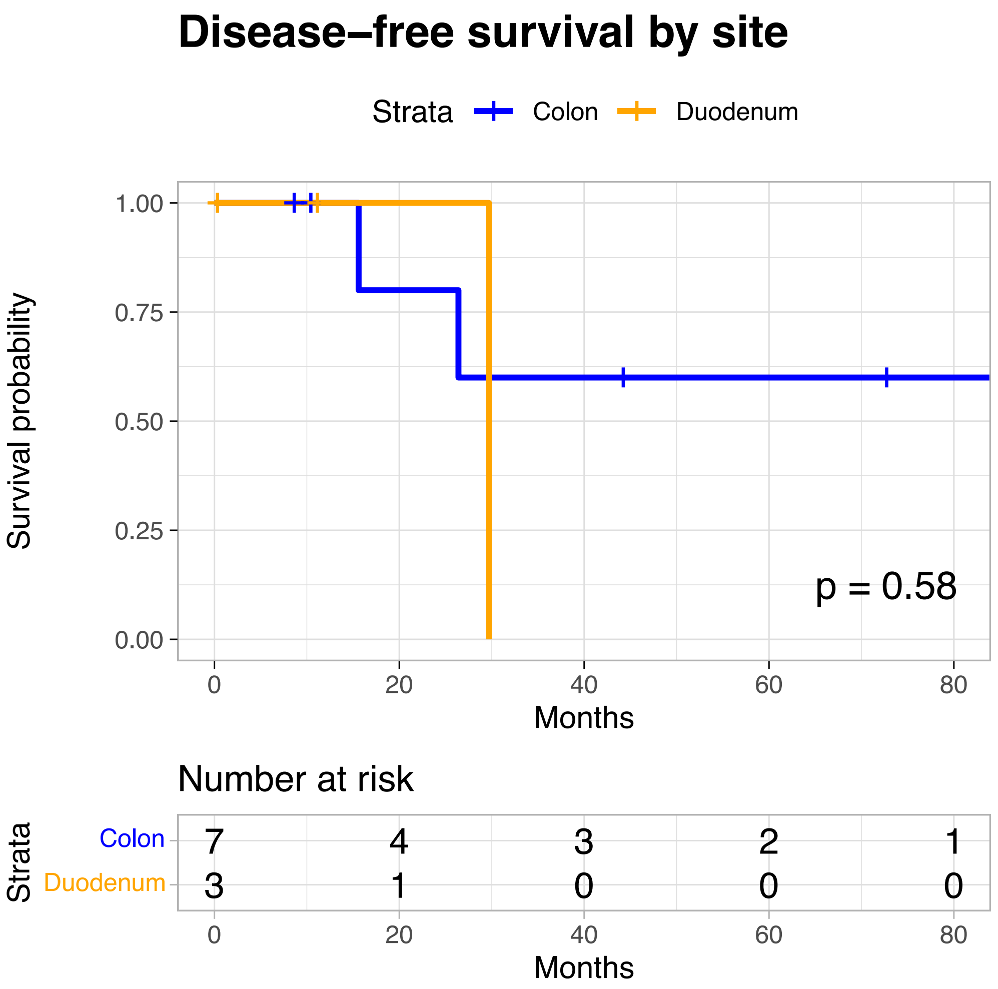
Disease–free survival by site
Strata Colon Duodenum
Survival probability
1.00
0.75
0.50
0.25
0.00
p = 0.58
0
20
40
60
80
Months
Number at risk
Strata
Colon 7 4 3 2 1
Duodenum 3 1 0 0 0
0
20
40
60
80
Months

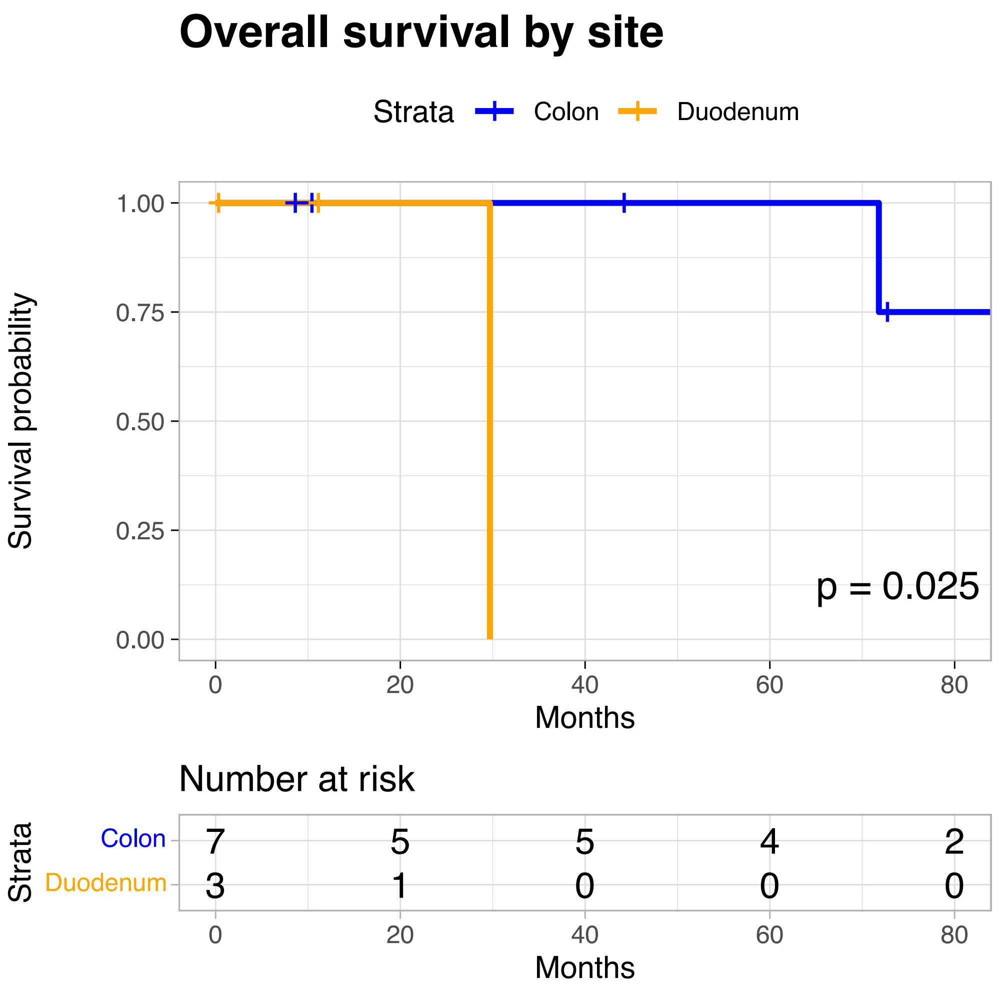
Overall survival by site
Strata Colon Duodenum
Survival probability
1.00
0.75
0.50
0.25
0.00
p = 0.025
0
20
40
60
80
Months
Number at risk
Strata
Colon 7 5 5 4 2
Duodenum 3 1 0 0 0
0
20
40
60
80
Months
