# Supplementary figures and images for: Pancreaticoduodenectomy with right hemicolectomy for advanced malignancy: a single UK hepatopancreaticobiliary centre experience
Source: Colorectal Dis. 2022 Sep 1;25(1):16–23. doi: 10.1111/codi.16303 (PMC10087186; doi:10.1111/codi.16303)

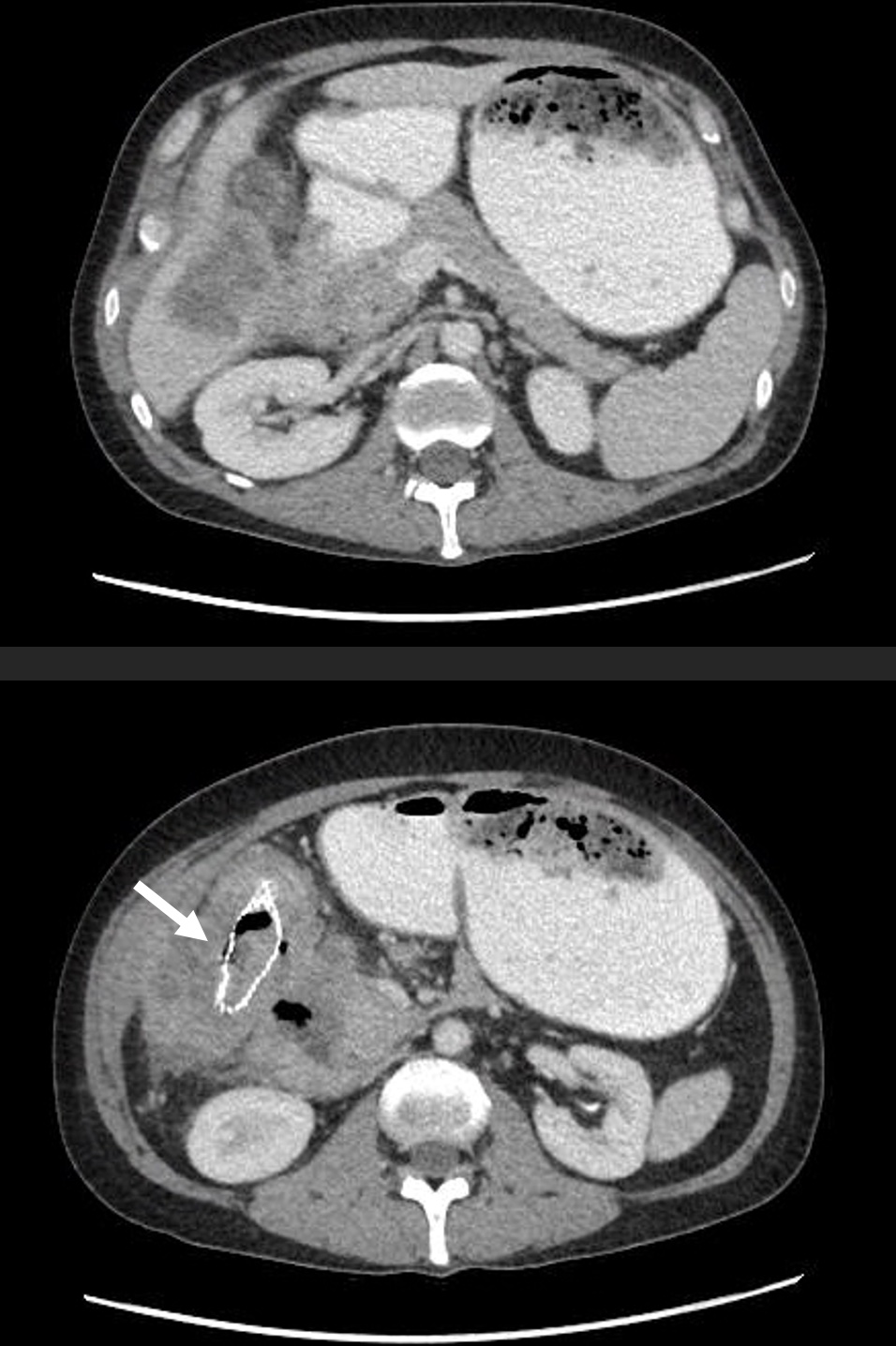

Supplement: Supplementary file 2 — Figure S2 [file CODI-25-16-s003.jpg]
