# Supplementary material for: Pancreaticoduodenectomy with right hemicolectomy for advanced malignancy: a single UK hepatopancreaticobiliary centre experience
Source: Colorectal Dis. 2022 Sep 1;25(1):16–23. doi: 10.1111/codi.16303 (PMC10087186; doi:10.1111/codi.16303)
Supplement: Supplementary file 3 — Table S1 [file CODI-25-16-s001.docx]

Supplementary Table 1: STROBE checklist

| **Item No.** | **Recommendation** | **Completed** | **Section** |
| --- | --- | --- | --- |
| 1 (a) | Indicate the study's design with a commonly used term in the abstract | Y | Abstract |
| 1 (b) | Provide in the abstract an informative and balanced summary of what was done and what was found | Y | Abstract |
| 2 | Explain the scientific background and rationale for the investigation being reported | Y | Background |
| 3 | State specific objectives, including any prespecified hypotheses | Y | Background |
| 4 | Present key elements of study design early in the paper | Y | Methods |
| 5 | Describe the setting, locations, and relevant dates | Y | Methods |
| 6 | Cohort study—Give the eligibility criteria, and the sources and methods of selection of participants | Y | Methods |
| 7 | Clearly define all outcomes, exposures, predictors, potential confounders, and effect modifiers | Y | Methods |
| 8 | For each variable of interest, give sources of data and details of methods of assessment (measurement) | Y | Methods |
| 9 | Describe any efforts to address potential sources of bias | Y | Methods |
| 10 | Explain how the study size was arrived at | Y | Methods |
| 11 | Explain how quantitative variables were handled in the analyses. | Y | Methods |
| 12 (a) | Describe all statistical methods, including those used to control for confounding | Y | Methods |
| 12 (b) | Describe any methods used to examine subgroups and interactions | Y | Methods |
| 12 (c) | Explain how missing data were addressed | N/A |  |
| 12 (d) | Cohort study—If applicable, explain how loss to follow-up was addressed | N/A |  |
| 12 (e) | Describe any sensitivity analyses | N/A |  |
| 13 (a) | Report numbers of individuals at each stage of study | Y | Results |
| 13 (b) | Give reasons for non-participation at each stage | N/A |  |
| 13 (c) | Consider use of a flow diagram | N/A |  |
| 14 (a) | Give characteristics of study participants (eg demographic, clinical, social) and information on exposures and potential confounders | Y | Results (Table 1) |
| 14 (b) | Indicate number of participants with missing data for each variable of interest | N/A |  |
| 14 (c) | Cohort study—Summarise follow-up time | Y | Results (Table 1) |
| 15 | Cohort study—Report numbers of outcome events or summary measures over time | Y | Results (Table 1) |
| 16 (a) | (a) Give unadjusted estimates and, if applicable, confounder-adjusted estimates and their precision (eg, 95% confidence interval) | Y | Results |
| 16 (b) | (b) Report category boundaries when continuous variables were categorized | Y | Results |
| 16 (c) | (c) If relevant, consider translating estimates of relative risk into absolute risk for a meaningful time period | N/A |  |
| 17 | Report other analyses done—eg analyses of subgroups and interactions, and sensitivity analyses | Y | Results |
| 18 | Summarise key results with reference to study objectives | Y | Discussion |
| 19 | Discuss limitations of the study, taking into account sources of potential bias or imprecision. Discuss both direction and magnitude of any potential bias | Y | Discussion |
| 20 | Give a cautious overall interpretation of results considering objectives, limitations, multiplicity of analyses, results from similar studies, and other relevant evidence | Y | Discussion |
| 21 | Discuss the generalisability (external validity) of the study results | Y | Discussion |
| 22 | Give the source of funding and the role of the funders for the present study and, if applicable, for the original study on which the present article is based | N/A |  |
